# Direct discharge for children with a greenstick or torus fracture of the wrist is a non-inferior satisfactory solution to traditional treatment

**DOI:** 10.1007/s00068-023-02391-w

**Published:** 2024-01-13

**Authors:** Jelle Friso Spierings, Gijs Johan Antoon Willinge, Henk Jan Schuijt, Diederik Pieter Johan Smeeing, Marike Cornelia Kokke, Joost Willem Colaris, Johan Carel Goslings, Bas Anne Twigt, Collaboration group, J. Ten Brinke, M. Leijnen, T. Nosewicz, D. L. Tiel Groenestege, R. N. van Veen

**Affiliations:** 1 Department of Trauma Surgery, St. Antonius Hospital, Soestwetering 1, 3543 AZ Utrecht, The Netherlands; 2 Department of Trauma Surgery, OLVG Hospital, Jan Tooropstraat 164, 1061 AE Amsterdam, The Netherlands; 3 Department of Orthopaedics and Sports Medicine, Erasmus University Medical Centre, Doctor Molewaterplein 40, 3015 GD Rotterdam, The Netherlands

**Keywords:** Torus fracture, Greenstick fracture, Soft cast, Removable orthosis, Direct discharge, Virtual fracture care, Efficiency

## Abstract

**Purpose:**

Direct Discharge protocols (DD) can alleviate strain on healthcare systems by reducing routine outpatient follow-up. These protocols include low-complex musculoskeletal injuries, such as isolated greenstick fractures or torus fractures of the wrist in children. While there is consensus on the effectiveness of DD, there is a lack of injury-specific powered studies. This study compares treatment satisfaction between DD and traditional treatment in children with a greenstick fracture or torus fractures of the wrist.

**Methods:**

Children with isolated torus or greenstick fractures of the distal radius or ulna were eligible for inclusion before (pre-DD cohort) and after (DD cohort) the implementation of DD in four hospitals. Traditionally, patients receive a (soft) cast and minimally one routine outpatient follow-up appointment. With DD, patients are discharged directly from the ED after receiving a brace and information, summarized in a smartphone app and a helpline for questions during recovery. The primary outcome was patient or proxy treatment satisfaction (0 to 10), and a power analysis was performed to assess non-inferiority. Secondary outcomes included complications, functional outcomes measured in Patient-Reported Outcomes Measurement Information System Upper Extremity (PROMIS UE), primary healthcare utilisation, and secondary healthcare utilisation (follow-up appointments and imaging).

**Results:**

In total, 274 consecutive children were included to analyse the primary endpoint. Of these, 160 (58%) were male with a median age of 11 years (IQR 8 to 12). Pre-DD and DD treatment satisfaction did not vary statistically significantly for greenstick fractures (*p* = 0.09) and torus fractures (*p* = 0.93). No complications were observed. PROMIS UE showed no statistically significant differences before and after implementation of direct discharge protocol for torus (*p* = 0.99) or greenstick (*p* = 0.45) fractures. Secondary healthcare utilisation regarding follow-up was significantly lower in the DD-torus cohort compared to the pre-DD torus cohort, with a mean difference (MD) of − 1.00 follow-up appointments (95% Confidence Interval (CI) − 0.92 to − 1.13). Similar results were found in the pre DD-greenstick cohort compared to the pre-DD-greenstick cohort (MD): − 1.17 follow-up appointments, 95% CI − 1.09 to − 1.26).

**Conclusion:**

Direct Discharge is non-inferior to traditional treatment in terms of treatment satisfaction for paediatric patients with greenstick or torus fractures of the wrist compared to children treated with rigid immobilisation and routine follow-up. Furthermore, the results demonstrate no complications, comparable functional outcomes, and a statistically significant reduction of secondary healthcare utilisation, making DD a good solution to cope with strained resources for children with an isolated greenstick fracture or torus fracture of the wrist.

## Introduction

Fractures of the wrist are among the most common injuries in paediatric patients, of which over 40% are low-complex, minor injuries [1]. Isolated torus and greenstick fractures without substantial displacement recover quickly and are most often treated with a cast [2, 3]. Low-complex paediatric wrist fractures, such as torus fractures or isolated greenstick fractures of the radius or ulna, have increasingly been managed through Direct Discharge protocols (DD) and Virtual Fracture Clinics (VFC) [4–7]. In its Dutch adaptation, DD involves discharging patients directly from the Emergency Department (ED) without routine follow-up, providing them with a removable orthosis or sling, and information through a mobile self-care application called the Virtual Fracture Care application (VFC app). Evidence supports DD’s overall safety and effectiveness as an alternative to traditional treatment protocols while reducing the need for secondary healthcare utilisation, resulting in comparable patient-reported outcomes (e.g. functional outcome and satisfaction scores) and little to no adverse outcomes (e.g. complications and persistent complaints) [7–9]. This method has gained popularity in the Netherlands, catalysed by the COVID-19 pandemic, and is currently standard care in over 30 of 80 Dutch hospitals. Although current evidence proves that the DD concept is satisfactory and safe, powered injury-specific results are lacking. Consequently, the optimal follow-up frequency for specific types of injuries remains unclear.

Therefore, the aim of this study was to determine whether treatment with a Direct Discharge protocol resulted in non-inferior child- and parental satisfaction in children with an isolated greenstick fracture or torus fracture of the wrist, compared to children treated with rigid immobilisation and routine follow-up.

## Methods

### Design

**Fig. 1:**
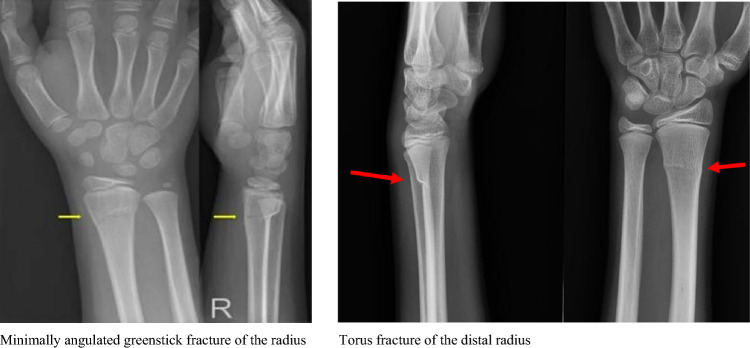
Examples of torus fractures and greenstick fractures of the distal radius included in the direct discharge protocol

This multicentre, prospective observational cohort study took place in four Dutch level 2 trauma teaching hospitals from the 1st of December 2021 to the 31st of August 2022, alongside the implementation of DD. Eligible patients had isolated torus or greenstick fractures of the distal radius or ulna with acceptable angulation according to the Dutch guideline based on age-related residual growth (Fig. 1) [10]. Furthermore, eligible patients had isolated injuries, low-energy trauma, Glasgow Coma Scale over 14, and spoke Dutch or English fluently. The study comprised two cohorts, a pre-DD cohort and a DD cohort, divided by the hospital implementation date. No comorbidity or cognitive impairment restrictions were applied. However, patients were excluded if healthcare professionals deemed DD unsuitable (e.g., social care reasons or language barrier) or if they received initial treatment in another hospital or requested follow-up in a hospital close to home.

#### Pre-DD (traditional treatment)

Before DD, patients were treated according to local trauma protocols, which varied between hospitals but often involved (soft) cast immobilisation for two to four weeks and verbal injury-related information at the ED. At least one outpatient follow-up appointment was scheduled for review, detailed information, and definitive management within 2 weeks after the injury.

#### Direct discharge protocol

Derived from the British Virtual Fracture Clinic (VFC) model, DD included 12 treatment protocols for low-complex musculoskeletal injuries, including greenstick- and torus fractures of the distal radius or ulna [10]. With DD, patients were discharged directly from the ED without routine follow-up. They received a removable orthosis instead of a cast and verbal information at the ED, which is summarised in a mobile self-care application, the VFC app. During the review of all ED cases the next working day, eligibility for DD was verified by an orthopaedic or trauma surgeon and radiologist. DD varies from traditional treatment in (1) the mode of routine outpatient follow-up (changing from physical to none, (2) the immobilisation (changing from a (soft) cast to removable orthosis), and (3) the delivery of uniform information (changing from verbal to digital).

### Outcomes

#### Satisfaction

The primary outcome of this study was satisfaction on a scale ranging from 0 to 100, 0 meaning very dissatisfied, to 100, meaning very satisfied, at three months after injury. Satisfaction was assessed in the three domains that change most with DD: overall treatment, immobilisation, and recovery information with patients or proxies (if patients were below the age of 12 years). All scores were rounded to numbers from one to ten to improve readability. The minimal clinically important difference (MCID) was set at 1.0 on a ten-point scale [6]. Treatment preferences were reported as face-to-face, neutral, or an application (such as the VFC app).

#### Immobilisation related outcomes

Immobilisation-related outcomes included ‘complications,’ ‘functional outcomes,’ and ‘analgesic use.’ Complications were evaluated by complication rate and type. Functional outcomes were measured with the Patient Reported Outcomes Measurement Information System for Upper Extremity (PROMIS UE) [7]. Currently, no musculoskeletal Patient Reported Outcome Measures are validated for children. To provide a holistic perspective of functional outcomes, relevant parameters of the CORE-kids outcomes have been used to provide additional data, including return to manual dexterity, return to sports, and return to activities of daily living (ADL) [11]. These were measured in four-point Likert scales ranging from 1, meaning complete return, to 4, no return at all. Additionally, analgesic use was measured in binary (yes/no), and by the type of analgesic used, including injury-site cooling, acetaminophen, and non-steroid anti-inflammatory drugs (NSAID). The timing of analgesic use was measured binary per week during the first four weeks (yes/no).

#### Healthcare utilisation

Healthcare utilisation was evaluated by ‘primary healthcare utilisation’ and ‘secondary healthcare utilisation’. Primary healthcare utilisation was evaluated by the number-, and frequency of injury-related follow-up appointments with a general practitioner or physiotherapist after the ED visit. Secondary healthcare utilisation was assessed based on the frequency of injury-related follow-up appointments and imaging procedures, including radiographs, computed tomography (CT), and magnetic resonance imaging (MRI).

#### VFC app use

VFC app use was evaluated by the frequency of use during the first 3 months of recovery as reported by patients or proxies.

### Recruitment and consent

After consent at the ED, eligible children or proxies were contacted by phone 3 months post-injury for a short survey, and an opt-out form was sent. Satisfaction scores, functional outcomes, and primary healthcare utilisation were collected, coded, and data were stored in an online database using RedCap [8]. The Electronic Patient Record was used to collect secondary healthcare utilisation and demographic data (age in years and sex) at least 3 months post-injury.

### Statistical analysis

Statistical analysis was performed using SPSS version 26.0.0.2 (IBM Corporation, Armonk, NY, United States) [12]. Normal distribution of continuous data was assessed with visual analysis of histograms, Kolmogorov and Shapiro–Wilk tests. Discrete variables were reported as numbers (percentages of the total population). The Chi-square test was used to determine the statistical significance of categorical variables. The Mann–Whitney *U* test was used to determine the statistical significance of non-parametric independent data. All reported *p-*values were 2-sided and considered statistically significant if they were lower than 0.05.

#### Power analysis

The primary outcome was treatment satisfaction at 3 months post-injury. This study aimed to demonstrate non-inferiority in satisfaction with traditional treatment compared to DD. A calculation was made based on the average satisfaction of 7.95 (standard deviation (SD) 1.7) on a 10-point scale in the pilot study [9]. Assuming a Minimal Clinically Important Difference (MCID) of 1.0 points on a 10-point scale, 90% power, one-sided *α* = 0.025, and an SD of 1.7, 124 patients (62 per arm) were required to show non-inferiority [6]. Allowing for a 10% loss to follow-up during the inclusion period, the minimum sample size was set at 136 per injury. This resulted in a total of 68 patients per injury per treatment type.

### Patient and public involvement

Patients were not involved in the design, intervention, research question, or outcome measures of this study.

### Ethics

The study was conducted under the Declaration of Helsinki and was approved by the local medical ethical review board Utrecht (W21.261). The institutions of the authors affiliated with St. Antonius Hospital and OLVG Hospital have received funding for support of this work by an unrestricted grant from the Dutch Organization for healthcare sciences and healthcare innovation, ZonMw, The Hague, The Netherlands, grant number: 516012524. None of the authors have a conflict of interest to declare for the execution of this study. Studydata is accessible upon request.

## Results

### Demographics

Between the 1st of December 2021 and the 31st of August 2022, 457 patients were screened. Of those, 267 (58%) were male. A total of 221 patients were treated with traditional treatment, of which 130 sustained a torus fracture and 91 a greenstick fracture. Treatment with DD was given to 236 patients, of whom 138 sustained a torus fracture and 98 a greenstick fracture. Study follow-up was completed on the 30th of November 2022, with collected primary outcomes of at least 68 patients per treatment group and a total of 274 patients. Baseline demographics were similar in both treatment groups (Table 1). During the recruitment period, over 1.4 times as many children presented to the ED with a torus fracture compared to a greenstick fracture in both the pre-DD cohort (130/91) and the DD cohort (138/98).

**Table 1:** Baseline characteristics of included patients with a greenstick- or torus fracture of the wrist in the evaluation of direct discharge compared to traditional treatment

	Torus fracture			Greenstick fracture		
	Pre-DD (*n* = 68)	DD (*n* = 68)	*p-*value	Pre-DD (*n* = 68)	DD (*n* = 70)	*p*-value
Age; median (IQR)	11 (7.8 to 12.3)	11 (8 to 13)	0.95	9 (6.5 to 12)	11 (8 to 13)	0.08
Gender male; *n* (%)	39 (57)	43 (63)	0.73	36 (53)	42 (60)	0.16

*IQR* Interquartile range, *DD* direct discharge

### Satisfaction

**Fig. 2:**
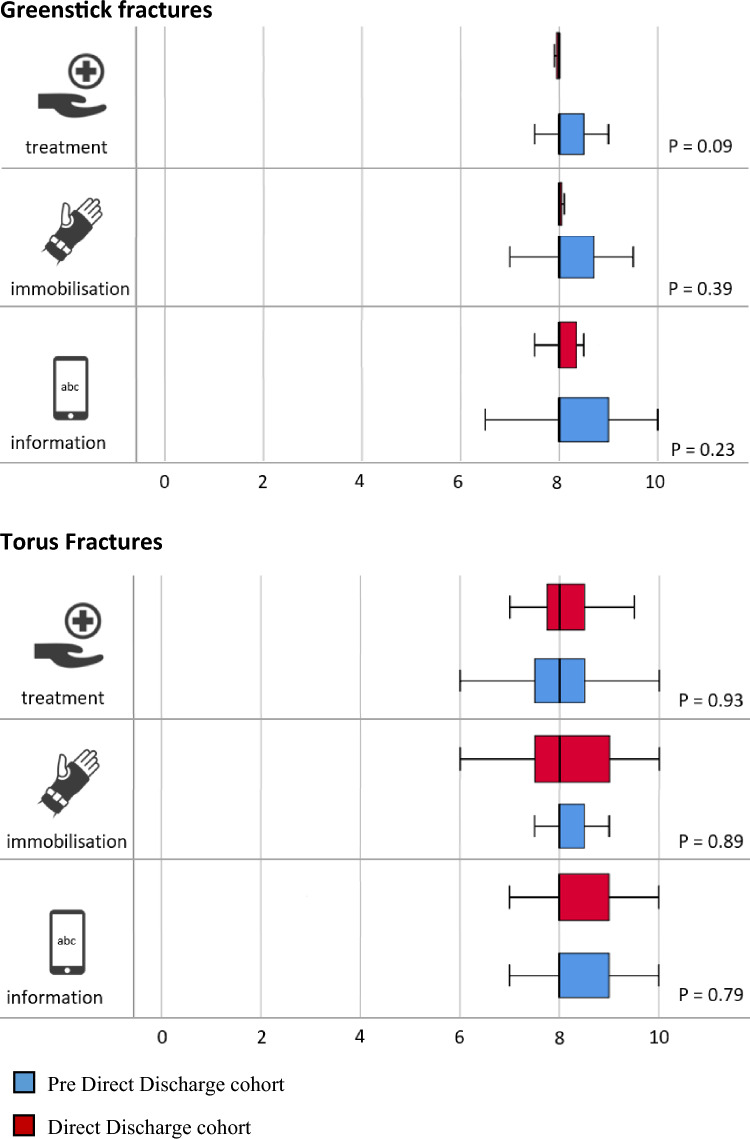
Box plot diagrams with median satisfaction scores on a 10-point scale of children with a greenstick or torus fracture of the wrist in the evaluation of the direct discharge protocol compared to traditional treatment

No statistically significant differences in treatment satisfaction were found between the pre-DD cohort (median 8.0, IQR 7.5 to 8.5) and the DD cohort for torus fractures (median 8.0, IQR 7.6 to 8.5) (*p* = 0.93). The same applied to pre-DD (median 8.0, IQR 8.0 to 8.5) and DD patients (median 8.0 IQR 7.9 to 8.0) (*p* = 0.09) with a greenstick fracture. Similar scores were observed for satisfaction with immobilisation for torus fractures pre-DD (median 8.0, IQR 8.0 to 8.5) and with DD (median 8.0, IQR 7.5 to 9.0) (*p* = 0.89), and greenstick fractures pre-DD (median 8.0, 8.0 to 8.8) and with DD (median 8.0 to 8.1) (*p* = 0.39), and satisfaction concerning information during recovery for torus fractures pre-DD (8.0, IQR 8.0 to 9.0) and with DD (8.0, IQR 8.0 to 9.0) (*p* = 0.79) and greenstick fractures pre-DD (median 8.0, IQR 8.0 to 9.0) and with DD (median 8.0, IQR 8.0 to 8.5) (*p* = 0.23) (Fig. 2). Among included patients and proxies in the pre-DD cohort, 89 (65%) preferred an application (such as the VFC app), 42 (31%) preferred face-to-face follow-up, and 5 (4%) had no preference.

### Immobilisation related outcomes

#### Complications

Patients who sustained a torus fracture in the pre-DD and DD cohort reported no complications. In the pre-DD cohort of patients with a greenstick fracture, 2/68 (3%) reported persistent pain three months after injury. Both patients were scheduled for an outpatient follow-up. After reassurance and information, no interventions were required. One patient (1/70, 1%) with a greenstick in the DD cohort reported skin irritation due to the removable orthosis. This patient switched from a removable orthosis to a soft cast to support the fracture, after which follow-up was completed.

#### Functional outcomes

PROMIS UE scored at 3 months post-injury did not differ significantly between pre-DD and DD patients in the torus-cohort (*p* = 0.99), and the greenstick cohort (*p* = 0.65) with a median score of 8.0 (IQR 8.0 to 8.0) in all groups (Table 2). Almost all patients with a torus fracture in the pre-DD group had a full return to previous manual dexterity, sports, and ADL, except one (1/68, 1%) who was somewhat limited in all three. In the DD group, all patients with a torus fracture had a full return to previous manual dexterity, sports, and one (1/68, 1%) was somewhat limited in return to ADL. All patients with greenstick fractures in the pre-DD group had a full return to previous manual dexterity. In the DD group, all patients had a full return of function, except two (2/68, 3%) who were somewhat limited in return to sports and one (1/68, 1%) who was somewhat limited in return to ADL (Table 2).

**Table 2:** Immobilisation related outcomes per treatment group in paediatric patients with an isolated greenstick- or torus fracture of the distal forearm

	Torus fracture		*p*-value	Greenstick fracture		*p-*value
	Pre-DD^a^ (*n* = 68)	DD^a^ (*n* = 68)		Pre-DD^a^ (*n* = 68)	DD^a^ (*n* = 70)	
Complications	0	0		2	1	
Persistent pain (VAS < 3)^c^	0	0		2	0	
Skin irritation	0	0		0	1	
Functional outcome						
Median PROMIS UE, from 8 to 40 (IQR)^f,b^	8.0 (8.0 to 8.0)	8.0 (8.0 to 8.0)	0.99	8.0 (8.0 to 8.0)	8.0 (8.0 to 8.0)	0.65
Return to manual dexterity						
Full return (%)	67 (99)	68 (100)		68 (100)	70 (100)	
Somewhat limited; *n* (%)	1 (1)	0 (0)		0 (0)	0 (0)	
Return to sports						
Full return; *n* (%)	67 (99)	68 (100)		68 (100)	70 (100)	
Somewhat limited (%)	1 (1)	0 (0)		0 (0)	0 (0)	
Return to ADL^h^						
Full return; *n* (%)	67 (99)	67 (99)		68 (100)	69 (99)	
Somewhat limited; *n* (%)	1 (1)	1 (1)		0 (0)	1 (1)	
Used analgesics during recovery (yes/total)	42 (62)	52 (76)		43 (63)	45 (64)	
Type of analgesic used						
Cooling	1	0		0	0	
Acetaminophen	39	52		43	44	
NSAID^d^	2	0		1^g^	1	
Timing of analgesic use						
First week	42	52		43	45	
Second week	2	4		2	6	
Third week	1	0		0	1	
Fourth week	0	0		0	1	
After fourth week	0	0		0	0	

^a^Direct discharge

^b^Interquartile range

^c^Visual analogue scale

^d^Non-steroidal anti-inflammatory drugs

^f^Patient reported outcomes measurement information system for upper extremity

^g^Patient used NSAID and acetaminophen

^h^Activities of daily living

#### Analgesic use

Analgesic use was high in the first week post-injury in all four groups and declined rapidly in the second week. Most pre-DD torus patients used analgesics (42/68, 62%), mostly acetaminophen (39/42, 93%), and some NSAIDs (2/42, 3%) or cooling (1/42, 1%). Most DD torus patients (52/68, 76%) used analgesics, all using acetaminophen. A similar number of pre-DD greenstick fracture patients (43/68, 63%) used analgesics, all using acetaminophen and one combining this with NSAIDs. Most DD greenstick patients 45/70 (64%) used analgesics, with 44/45 (98%) using acetaminophen and one using NSAIDs (Table 2).

### Healthcare utilisation

#### Primary healthcare utilisation

In the pre-DD and DD cohort, four patients with a torus fracture utilised primary healthcare. Four patients with a greenstick fracture in the pre-DD cohort used primary healthcare during recovery, and six patients in the DD cohort, of which two patients visited both the physiotherapist and general practitioner (Table 3).

**Table 3:** Healthcare utilisation and preferences per treatment group in paediatric patients with an isolated greenstick- or torus fracture of the distal forearm

	Torus fracture		Greenstick fracture	
	Pre-DD (*n* = 68)	DD (*n* = 68)	Pre-DD (*n* = 68)	DD (*n* = 70)
Primary healthcare utilisation				
General practitioner	1	3	4	4
Physiotherapist	3	1	0	4
Secondary healthcare utilisation				
Face-to-face follow-up				
0	6	59	7	59
1	47	7	37	10
2	16	2	27	1
> 2	0	0	0	0
Follow-up by phone				
0	64	68	67	70
1	4	0	1	0
2	0	0	0	0
> 2	0	0	0	0
Additional imaging				
X-ray	1	0	3	1
CT-scan	0	0	0	0
MRI-scan	0	0	0	0

*DD* Direct discharge, *CT* computed tomography, *MRI* magnetic resonance imaging

#### Secondary healthcare utilisation

The mean number of secondary healthcare appointments in the pre-DD cohort of torus fractures was 1.16 (SD 0.54) versus 0.16 (SD 0.47) in the DD cohort, resulting in a mean difference (MD) of 1.00 (95% CI 0.92 to 1.13). The mean number of secondary healthcare appointments in the pre-DD cohort of patients with a greenstick fracture was 1.34 (SD 0.64) versus 0.17 (0.39) in the DD cohort, with an MD of 1.17 (95% CI 1.09 to 1.26). Imaging was comparable and low in all four groups (Table 3).

### VFC app use

The mean frequency of application use during recovery was 2.1 (SD 2.2) times during recovery in patients with a torus fracture and 1.5 (SD 2.3) times during recovery in patients with a greenstick fracture.

## Discussion

Results of this prospective multicentre cohort study showed that DD is non-inferior in terms of satisfaction regarding overall treatment, immobilisation, and information during the recovery process compared to traditional treatment for patients with torus fracture or an isolated greenstick fracture of the distal forearm, compared to patients treated with rigid immobilisation and routine follow-up.

The satisfaction results of this study are in line with the current literature. In previous underpowered studies with cohorts comparable to the pre-DD and DD cohort, treatment and immobilisation satisfaction levels were similarly high [13–15]. Comparable to other studies, a removable orthosis with immediate discharge was preferred over a cast with routine follow-up [3, 14]. One study reported the preference for a cast on day one post-injury due to pain [3]. However, this preference was not observed in the study at 6 weeks or after, showing no differences in satisfaction scores or pain scores for both treatment methods. Furthermore, in this study, high levels of perceived safety and the preference for DD and a self-care application, such as the VFC app, over face-to-face follow-up for patients with torus- or greenstick fractures of the wrist were mentioned. These parameters have been reported in eHealth evaluation studies but are a novelty in DD and VFCs [16].

Similar to the literature, a low amount of complications and good functional outcomes were detected in both cohorts for both injuries individually, indicating comparable results between traditional treatment and DD [3, 9]. Both PROMIS UE and the parameters of the CORE kids score, were comparably high, indicating good functional outcomes of these injuries at three months follow-up. Analgesic use was comparable to previous studies, showing high levels of analgesic use in the first week, followed by a rapid decline showing limited analgesic requirement throughout recovery, further strengthening the limited need for analgesics for these injuries and immobilisation types [3]. The treatment of torus fractures with less rigid immobilisation than a cast is supported by current literature, as these are widely regarded as stable, self-limiting fractures [3, 13, 14, 17]. This has been less evident for greenstick fractures, with study results reporting further displacement during the first 14 days after manually realigned greenstick fractures [17]. However, this secondary displacement never resulted in a change in fracture management, and this study excluded realigned fractures [17]. The lack of surgical intervention might be explained by the excellent remodelling capacities of distal epiphysis of the radius, which is the most active epiphysis in the growing child [18].

Based on the low complications, good functional outcome, and satisfactory results with immobilisation, we believe treatment with DD is safe. The statistically significant reductions in secondary healthcare utilisation were consistent with previous research and estimations [5, 19–21]. This shows that treatment of both fracture types can safely be reduced to one ED visit without negatively influencing satisfaction. The findings could make treatment strategies for greenstick fractures more uniform between hospitals regarding follow-up and immobilisation materials whilst reducing secondary healthcare utilisation.

This study has several strengths. First, the comprehensive outcomes provided a holistic perspective of patient satisfaction, functional outcome and treatment safety. Second, the study was adequately powered to detect an MCID in satisfaction with a representable study population. Third, the pragmatic pre-post multicentre design alongside the implementation of DD provided a cheap, effective, and quick alternative to often time-consuming and often expensive RCTs with an acceptable level of evidence. This study also has limitations. Only patients who responded to the phone call were included in the study, potentially leading to selection bias. Furthermore, the timing of functional outcome at 3 months follow-up might have been suboptimal to measure functional outcome, as almost all patients had fully recovered by then. Nevertheless, all scores were high with both protocols.

The outcomes of this study, along with previously published studies, can be used to support the implementation of DD as the new standard of care for specific injuries, which could support the reduction of secondary healthcare utilisation whilst maintaining non-inferior treatment satisfaction. Furthermore, DD reduces the heterogeneity in treatment of these low-complex injuries [22]. The study design used can be applied to rapidly and comprehensively assess differences between treatment options without hindering implementation in interested hospitals. A central registry with uniform parameters could be considered to improve DD further and potentially include new injuries in the protocol. Future studies should focus on the optimal timing to measure functional outcomes in paediatric patients and the validation of PROMs in children. Our future efforts will be focused on developing similar protocols for other and also more complex injuries to alleviate the burden on healthcare systems already under strain.

To conclude, the results of this multicentre cohort study on children with a greenstick fracture or torus fractures of the wrist. showed that Direct Discharge is non-inferior in terms of satisfaction with treatment, satisfaction with immobilisation, and satisfaction with information during the recovery process compared to traditional treatment. These results could help de-medicalise these injuries and prevent the overuse of healthcare resources for these common fractures.

## Data Availability

Studydata is accessible upon request.
